# Alteration of actin dependent signaling pathways associated with membrane microdomains in hyperlipidemia

**DOI:** 10.1186/s12953-015-0087-0

**Published:** 2015-12-01

**Authors:** Viorel-Iulian Suica, Elena Uyy, Raluca Maria Boteanu, Luminita Ivan, Felicia Antohe

**Affiliations:** Institute of Cellular Biology and Pathology “Nicolae Simionescu”, 8 BP Hasdeu Street, PO Box 35–14, 050568 Bucharest, Romania

**Keywords:** Detergent resistant membrane microdomains, Hyperlipidemia, Actin cytoskeleton, Proteomics, Mass spectrometry

## Abstract

**Background:**

Membrane microdomains represent dynamic membrane nano-assemblies enriched in signaling molecules suggesting their active involvement in not only physiological but also pathological molecular processes. The hyperlipidemic stress is a major risk factor of atherosclerosis, but its exact mechanisms of action at the membrane microdomains level remain elusive. The aim of the present study was to determine whether membrane-cytoskeleton proteome in the pulmonary tissue could be modulated by the hyperlipidemic stress, a major risk factor of atherosclerosis.

**Results:**

High resolution mass spectrometry based proteomics analysis was performed for detergent resistant membrane microdomains isolated from lung homogenates of control, ApoE deficient and statin treated ApoE deficient mice. The findings of the study allowed the identification with high confidence of 1925 proteins, 291 of which were found significantly altered by the modified genetic background, by the statin treatment or both conditions. Principal component analysis revealed a proximal partitioning of the biological replicates, but also a distinct spatial scattering of the sample groups, highlighting different quantitative profiles. The statistical significant over-representation of *Regulation of actin cytoskeleton*, *Focal adhesion* and *Adherens junction* Kyoto Encyclopedia of Genes and Genomes signaling pathways was demonstrated through bioinformatics analysis. The three inter-relation maps comprised 29 of regulated proteins, proving membrane-cytoskeleton coupling targeting and alteration by hyperlipidemia and/or statin treatment.

**Conclusions:**

The findings of the study allowed the identification with high confidence of the main proteins modulated by the hyperlipidemic stress involved in the actin-dependent pathways. Our study provides the basis for future work probing how the protein activities at the membrane-cytoskeleton interface are dependent upon genetic induced hyperlipidemia.

**Electronic supplementary material:**

The online version of this article (doi:10.1186/s12953-015-0087-0) contains supplementary material, which is available to authorized users.

## Background

Atherosclerosis is a multi-factorial chronic disease, which constitutes one of the leading causes of death mainly in developed countries. Although for a long time this pathology was considered a slow and irreparable process, recent development has provided important evidence that it is a multi-zonal, dynamic and when early treated reversible process [1]. Endothelial cells are especially implicated in the development and progression (or regression) of the atherosclerotic lesions, being the first cell monolayer insulted by plasma biochemical changes, undergoing inflammatory activation in response to atherogenic stimuli such as modified lipoproteins that accumulate in the arterial wall generating atherosclerotic plaques [2, 3].

The pulmonary endothelium can be viewed as a discrete organ regulating important functions, such as exchange of solutes, modulation of vascular tone, control of homeostasis, fibrinolysis, coagulation, regulation of vasculogenesis and angiogenesis, interaction with platelets and leukocytes [4]. Although atherosclerotic plaques do not usually develop in pulmonary vasculature, it has been stated that the pulmonary endothelium in particular serves as a biological determinant that can be modulated for health improvement using angiotensin converting enzyme inhibitors and statins [5, 6]. Published data demonstrated that atherosclerotic stress factors such as high-fat diet [7, 8], hypertension [9], reactive oxygen intermediates [10], excessive NO production [11], overproduction of pro-inflammatory cytokines, chemokines [12, 13] and deregulation of coagulation and fibrinolysis [14, 15], can activate the pulmonary endothelium, leading to distortion of multiple signaling pathways, with significant impact on the stability of atherosclerotic plaques located in the lesion-prone area of the vascular tree. All these data are directly correlated with the well-established clinical profile of patients with advanced atherosclerosis showing recurrent pulmonary diseases and insufficient oxygenation associated with general fatigue and limited physical efforts.

Detergent resistant membrane (DRM) microdomains are small (10–200 nm), heterogeneous and dynamic cholesterol and sphingolipid enriched domains that compartmentalize cellular processes, such as cholesterol homeostasis, and endocytosis [16]. Smaller DRM microdomains can coalesce together to form larger platforms through protein-protein and protein-lipid interactions [17]. For a long time, DRM microdomains, owing their name to the classical procedure of extraction in non-ionic detergents and flotation in a sucrose gradient [18], have been associated with cell signaling [19, 20]. Their proteomic profile, enriched in signaling proteins such as heterotrimeric G proteins, non-receptor tyrosine kinases and protein phosphatases [21–23], suggested the active involvement in the molecular mechanisms that control both the physiologic and the pathologic cellular processes.

Along with the cellular junctions and glycocalyx coat, the endothelial cell cytoskeleton plays an important role in maintaining cellular structural integrity and signal transduction in response to mechanical forces [24–26].

Previous studies demonstrated that under turbulent flow, endothelial dysfunction is favored through cytoskeletal remodeling, promoting atherosclerotic processes by endothelial architecture alteration [27]. It is also well established that inflammation of the vascular bed is associated with endothelial cytoskeletal redistribution, which leads to an increase in intercellular gap size and paracellular permeability [28]. Closely associated with signaling molecules, cytoskeletal and adhesion molecules are routinely found in DRM preparation, such as actin, myosin, vinculin, cofilin, cadherin, filamin, ezrin, etc. [22, 29, 30]. The presence of cytoskeletal proteins in the DRM microdomains proteome is an indication that these microdomains actively interact with the cytoskeleton, providing the needed energy and the stability for the proper function of aggregated membrane microdomain structures and signaling pathways [31].

Statins are lipid-lowering drugs that were developed and tested clinically on the basis of their properties to suppress cholesterol biosynthesis. They work by selectively and competitively inhibiting 3-hidroxy-3-methyl-glutaryl-coenzyme A (HMG-CoA) reductase and by promoting the up-regulation of low density lipoproteins-cholesterol receptors on the plasma membrane [32, 33]. Various measurements of haemostatic parameters provide further demonstration for the beneficial effects of statins on endothelial cells, including promotion of a pro-fibrinolytic state [34]. Also, fluvastatin has been shown to inhibit matrix metalloproteinase-1 expression and oxidative damage in vascular endothelial cells, thus improving endothelial dysfunction associated with atherosclerosis [35, 36].

ApoE deficient mice develop spontaneous atherosclerotic lesions, even on a low fat chow diet [37, 38]. This model was generated from C57BL6 mice by knocking out the ApoE gene leading to Apo E deficient animals that showed impaired clearing of plasma lipoproteins and development of atherosclerosis. In a short time, they exhibit extremely high level of plasma cholesterol and triglycerides and more advanced aortic lesions than the fatty streaks observed in normal inbred strains [39].

In the present study we added a supplementary stress factor, the high fat diet to accelerate the development of atherosclerotic lesions in ApoE knockout (ApoE KO) mouse experimental model [40, 41]. The effect of statin therapy was monitored in a similar lot of animals. The designed workflow took advantage of the well-established methodology to isolate DRM microdomains from lung tissue of ApoE deficient mice to investigate the molecular mechanisms specifically modified in atherosclerosis. The high performance liquid chromatography tandem mass spectrometry approach and appropriate proteomic bioinformatics analysis were used. In the end, the evaluation of DRM microdomains proteome with or without statin treatment was performed with regard to the representative protein classes of actin-dependent signal transduction pathways, namely: *Regulation of Actin Cytoskeleton*, *Focal Adhesion* and *Adherence Junctions*. The proteomic study revealed a panel of differentially expressed proteins that play pivotal roles in the molecular mechanisms of membrane-cytoskeleton interactions in atherosclerosis. The selective proteins regulation by the statin treatment was evaluated. The results were also supported by the immunological validation method that was applied for some of these proteins.

## Methods

### Reagents and consumables

All chemicals used in this study were of electrophoresis, liquid chromatography or mass spectrometry grade, unless otherwise specified. Sodium fluvastatin was purchased from Novartis (Basel, Switzerland); MES [2-(N-Morpholino)ethanesulfonic acid], sodium chloride, urea, sodium deoxycholate (DOC), trizma hydrochloride, DL-dithiothreitol (DTT), iodoacetamide (IAA), N-acetyl-L-cysteine (NAC), ammonium bicarbonate, bicinchonic acid, Bradford reagent and all solvents were provided by Sigma-Aldrich (Missouri, USA). Sequencing grade modified trypsin was offered by Promega (Wisconsin, USA). Protease inhibitor cocktail Complete tablets was purchased from Roche (Indiana, USA). C18 solid phase extraction columns were acquired from Waters (Massachusetts, USA). Western blot detection was performed using Enhanced chemiluminescence plus reagent (GE Healthcare, Little Chalfont, UK). Mouse anti-human caveolin-1 monoclonal antibody, lot: 64023, clone: C060, catalogue number: 610058, BD Transduction Laboratories (Kentucky, USA), rabbit anti-mouse polymerase I and transcript release factor (PTRF) polyclonal antibody, lot: GR7161-2, catalogue number: ab48824 and mouse anti-beta actin, catalogue number ab6276, clone AC-15 antibody from Abcam (Cambridge, UK). Monoclonal anti-vinculin FITC, clone hVIN-1, catalogue number F7053 and IgG-HRP secondary antibodies either anti-mouse or anti-rabbit IgG peroxidase conjugate from Sigma-Aldrich (Missouri, USA) were used for immunoblotting experiments.

### Experimental animal models

Healthy 6 weeks male laboratory mice (Mus musculus) were used in the current study. The lot comprised a Black C57 control group (C, *n* = 3) fed with standard diet, a group of hyperlipidemic ApoE KO mice with the same genetic background as the control group (A, *n* = 3), that received four weeks a high fat diet (1 % cholesterol and 15 % butter) and a similar statin treated group of ApoE KO mice (At, *n* = 3) that after the four weeks of high fat diet and installation of atherosclerosis were transferred to standard diet together with oral gavages of fluvastatin sodium (10 mg/kg body/day) for another two weeks. The statin treatment was initiated together with starting of the low fat diet similar with the clinical practice, allowing only the genetic factor to act during statin administration. Thus, after two weeks of statin therapy the animals were compared with the four weeks atherosclerosis animals (group A) to clearly evidence the effect of imposed standard diet associated with low cholesterol medication.

The mice were kept in the animal husbandry facility under 12 h light/dark cycles with free access to food and water. All animal experiments were conducted in accordance with “International Guiding Principles for Biomedical Research Involving Animals” (Council for the International Organizations of Medical Sciences, December 2012) and Romanian Law no. 471/2002. The protocol was approved by the Ethic Committee of ICBP “N. Simionescu” (Permit Number: 373).

### Detergent resistant membrane microdomains isolation

DRM microdomains fractions were prepared as previously described [18]. Briefly, 200 mg of lung tissue fragments were solubilized on ice in 1.8 ml MES-buffered saline (MBS), pH 6.5 (containing: 25 mM MES, 0.15 M NaCl) and 1 % Triton X-100 with the use of a rotor-stator mechanical homogenizer (5 min at high speed). The resulting lysate was adjusted to 40 % sucrose with 2 ml of 80 % sucrose in MBS and placed on the bottom of an ultracentrifuge tube. A discontinuous sucrose gradient was formed by gently overlaying 4 ml of 30 %, followed by another 4 ml of 5 % sucrose in MBS. The sucrose gradient tubes were placed in the SW-41 rotor and centrifuged at 200,000 × g, for 19 h at 4 °C, using the Optima LE-80 ultracentrifuge (Beckman-Coulter, Fullerton, USA). Fractions (*n* = 12) were harvested from top to bottom, for biochemical determinations and afterwards experiments. Protein quantification was performed using bicinchoninic acid while cholesterol level determination was realized using the CHOD-PAP method (cholesterol assay kit, DIALAB GMBH, Neudorf, Austria). To validate the significant enrichment of the samples with endothelial plasma membrane the angiotensin I converting enzyme (ACE) activity was evaluated using Hip-L-His-L-Leu substrate, as previously described [42]. For each individual replicate, the two fractions (4 and 5) enriched in protein and cholesterol, with high ACE activity, were combined and diluted 5 times in MBS before a 4 h ultracentrifugation (200,000 × g at 4 °C). The resulting supernatant was removed and the pellet of each biological replicate was stored at −80 °C and analyzed separately for proper statistical significance evaluation.

### SDS-PAGE and immunoblotting assay

Equivalent amounts of protein from the collected fractions were separated by 12.5 % sodium dodecyl sulfate/polyacrylamide gel electrophoresis. The separated proteins were silver stained and the gel image acquisition was performed using ArtixScan 1100 scanner (Microtek, Hsinchu, Taiwan). The proteins from similar gels were transferred to nitrocellulose membranes and analyzed by Western Blot assay. The nonspecific binding was blocked with 5 % bovine serum albumin (BSA) in Tris-buffered saline (TBS) containing 0.05 % Tween 20, pH 7.6. The blots were than exposed for 2 h to the primary anti-caveolin-1 (0.25 μg/ml working concentration), anti-PTRF (1 μg/ml final concentration), or anti-beta actin (0.5 μg/ml working concentration) antibodies in TBS with 1 % BSA. Incubation for 1 h with appropriate secondary IgG-HRP antibodies was performed and the chemiluminiscence reaction was recorded. In the case of the vinculin antigen detection, only the first monoclonal antibody (conjugated with FITC) was used. For this situation, the specific excitation/emission FITC filters were used inside the Typhoon 9500 laser scanner (GE Healthcare, Uppsala, Sweden).

### Preparation of DRMs for mass spectrometric analysis

Sample solubilization was conducted in a highly denaturant buffer containing 8 M urea (as main chaotropic agent), 1 % sodium deoxycholate (DOC) and 0.1 % Tris–HCl (pH 8.8). The solubilization was conducted through powerful vortexing for 30 min on ice, followed by occasional shakeup for another 3 h on ice. The protein quantification was performed using the Bradford reagent and a bovine serum albumin 5 point standard curve (0.1-1 μg/μl). The sample was cleaned-up for lipids and salts by precipitation with methanol/chloroform/water (4:2:4) combination. The cysteine residues were reduced in freshly prepared denaturant buffer, pH 8.8, (containing 8 M urea, 0.1 M Tris–HCl, 0.1 mM EDTA and 20 mM DTT) for 60 min. Alkylation of the reduced proteins was conducted using 80 mM IAA in 0.1 M Tris–HCl and 0.1 mM EDTA buffer, for 90 min followed by quenching with 80 mM NAC in 0.1 M Tris–HCl and 0.1 mM EDTA buffer, for 30 min. All these steps were performed in the dark, under continuous stirring at room temperature. Before the digestion process, the sample buffer was diluted up to 1 M urea using 50 mM ammonium bicarbonate (pH 8.8) and DOC was added up to 1 % final concentration.

Proteolysis was performed overnight, at 37 °C, with stirring, using a 1:20 enzyme to substrate quantity ratio, using sequencing grade modified trypsin. After 14 h, the resulted peptide mixtures were acidified to pH 2–3 with formic acid for trypsin activity inhibition and DOC precipitation. DOC was discarded following a 20 min, 20,000 × g, at room temperature centrifugation. The desalting step was conducted using Sep Pek C18 columns. The purified peptides were eluted using 0.1 % formic acid in 80 % acetonitrile. The peptides were dried using the Concentrator plus system (Eppendorf, Hamburg, Germany) and stored at −80 °C until LC-MS analysis. Prior to the LC separation, the peptides were resuspended in 0.1 % formic acid, 5 % acetonitrile solution to final concentration of 0.5 μg/μl, using an ultrasonication bath (15 min).

### Tandem LC-MS analysis

LC-MS/MS experiments were performed using the Ultimate 3000 RSLC nano system (Dionex, California, USA) coupled to the LTQ Orbitrap Velos hybrid mass spectrometer (Thermo Scientific, California, USA). For each analysis, the sample (1 μl) was loaded in triplicate, into an Acclaim PepMap 2 cm × 75 μm i.d., C18, 3 μm, 100 A trap column (Dionex). The trap column was connected to the Acclaim PepMap RSLC 15 cm × 75 μm i.d., C18, 2 μm, 100 A analytical column (Dionex, California, USA). Solvent A was LC-MS grade water with 0.1 % (v/v) formic acid, and solvent B was represented by LC-MS grade acetonitrile with 0.1 % (v/v) formic acid. After washing the trap column for 3 min, peptides were eluted with a gradient of 2–35 % solvent B over 48 min (70 min total chromatographic method and MS acquisition) at 300 nl/min flow rate. Dynamic nano-electrospray source housing was utilized with uncoated SilicaTips, 12 cm length, 360 μm outer diameter, 20 μm inner diameter and 10 μm tip inner diameter. For ionization, 1500 V of liquid junction voltage and 250 °C capillary temperature were used. The mass spectrometer was operated in a top 6 data-dependent configuration at 60 k resolving power for full scan, with monoisotopic precursor selection enabled and mass correction by using lock mass, across the 300–2000 m/z domain. The analyses were carried out with collision induced dissociation (CID) fragmentation mode (with the m/z width of precursor window set to 2 and normalised collision energy of 35). The instrument operating software was Xcalibur 2.1.0 QF03489 build 1140 and LTQ Orbitrap Velos MS 2.6.0 build 1050. All liquid chromatography and mass spectrometry experiments parameters are provided as Additional file 1. 

### Database protein identification

Protein identification was performed using Proteome Discoverer 1.4 (Thermo Scientific, California, USA). The search engine was Mascot 2.4.1 (Matrix Science, London, UK) and the taxonomy was set on Mus musculus organism in UniProtKB/SwissProt fasta database, build 04.2013. A maximum of 2 missed cleavage sites were allowed. A mass tolerance for the precursor was set on 10 ppm and for the fragment on 0.8 Da. Oxidation of methionine and deamidation of asparagine and glutamine were enabled as dynamic modifications while carbamidomethylation of cysteine was set as fixed modification. The search workflow contained also a Percolator validation node [43] using a decoy database search with a *FDR* target lower than 0.05. The validation was based on the *q-value*. Proteins identified with only one peptide were verified manually. Proteome Discoverer Deamon 1.4 was utilized for performing raw file combination of replicate samples as well as batch searches for each biological condition.

### Label-free quantification

The label free relative quantification on the precursor level was performed with SIEVE 2.1 software (Thermo Scientific) that aligns MS spectra over retention time for different experimental conditions and detects frames that change along the different biological and technical replicates [44]. The parameters selected for alignment and frame detection needed for abundance calculation were as follows: MZ start: 300; MZ stop: 2000; PCA Process: force calculation; RT start: 0.01; RT stop: 69.99; alignment bypass: false; alignment min intensity: 1000; correlation bin width: 1; max RT shift: 0.2; tile size: 300; frames from MS2 scan: true; MZ width ppm: 10; RT width: 1.

The chromatographic alignment was founded on a scalable adaptive tiled algorithm, in which pairs of full scan spectra were compared and the spectra were then separated into bins of equal size. Then, a correlation between the two spectra (from each raw file) was calculated and the spectrum to spectrum correlations were used to construct a matrix. An optimal path through the matrix was determined, overlapping tiles were constructed continuing the path and when the full plane was tiled, a final alignment score was calculated, where a value of 1 represents a perfect alignment, which is only possible for the reference file chosen for the alignment. The ion score for peptide rescoring criteria was set at minimum 2. For minimization of technical errors, we performed normalization on the frame level, using the total ion chromatogram (TIC) normalization method, where the ion current was tallied between RT start (0.01) and RT stop (69.99). The calculation process derived from variance weighting, in which ratios were combined using 1/variance as weighting factor, where variance is the square of the standard deviation of the ratio. The filtering formula for detecting relevant differences among the distinct biological groups included the following parameters: a coefficient of variation below 30 % for all replicates, the fragmentation set to be performed only on the C12 monoisotopic peak (PRELEMENT = 0) and the detected frames to be associated with an identified peptide (GOOD ID = 1). The frames were manually checked for proper peak shape and overlapping of extracted ion chromatograms. After SIEVE filtering process, a cut-off of significance (*P* < 0.05) and ratio threshold (1.5 fold up- or down-regulation) was set at the protein level.

### Data mining, protein annotation and pathway analysis

General and detailed description of the various properties of proteins from our data and annotation based on gene ontology comparison (cellular component, molecular function and biological process) were obtained using a trial version of Protein Center software (Thermo Scientific). Protein Center databases were checked for protein quantity alterations and to match qualitative evaluation, integrate and project quantitative data onto over-represented *Kyoto Encyclopedia of Genes and Genomes* (KEGG) signaling pathways, publically available, online pathway database of molecular interactions from within specific organisms, pioneered by Kanehisa and his colaborators [45]. To estimate if a certain category of feature was disproportionately represented in a data set, statistical tests were carried out for a subset of interesting proteins (differentially expressed proteins from the A and At category vs. the C group). A statistical correction was used in Protein Center analysis which was based on the method suggested earlier [46]. This method corrects *P-values* based on the False Discovery Rate (FDR). Thus, a significance FDR level of 5 was chosen for the comparison of our differentially expressed proteins with the Mus musculus proteome reference set and an over-represented KEGG pathway map was found significant if an FDR *P-value* was below 0.05.

## Results

### Atherosclerosis animal model

For the acceleration of the onset and development of atherosclerotic plaques, the ApoE KO mice (A group) received a hyperlipidemic diet. Indeed as previously published, introducing the high fat diet results in development of the atherosclerotic lesion about seven weeks sooner when compared to the standard fed ApoE KO mice [39, 40]. In the present study, the body weights of the hyperlipidemic (A) (20.8 ± 0.24 g) as well as the statin treated hyperlipidemic group (At), (20.2 ± 0.22 g) remained almost constant, without any statistical significant changes, when compared to control (C) group (20.1 ± 0.38 g). However, the serum cholesterol (461.57 ± 49.39 mg/dl) and triglyceride (125.67 ± 10.19 mg/dl) levels were statistically increased (*P* < 0.001) in the A group when compared to those of the control, in which the serum cholesterol and triglyceride levels were 65.85 ± 1.56 mg/dl and 46.42 ± 1.85 mg/dl respectively. As expected, the statin treatment significantly reduced both the cholesterol (109.11 ± 14.65 mg/dl) and triglyceride levels (54.02 ± 0.91 mg/dl) of the At group to levels comparable to those in the control C. Since it is impossible to compare the statin effect in the same animals, we did evaluate the two weeks statin administration outcome with the atherosclerotic status reached by the animals with genetic and high fat diet induced hyperlipidemia (A group), just before the statin therapy. The described assessment closely matches the human patient situation receiving the statin administration together with low fat diet recommendation and may reveal clear evidence of the cholesterol lowering drug effect in the atherosclerotic committed animals (ApoE deficient mice), irrespective of the received diet.

Previously, our published data showed that experimental A group developed substantial atherosclerotic lesions in the proximal aorta and valves [7]. The mild fatty streaks developed by animals in the At group (statin treated) were found in good correlation with the serum lipid levels, similar to those in our current At experimental group, documenting that the statin therapy, as expected, will most likely delayed the development of the atherosclerotic plaques [7].

### Characterization of isolated DRM microdomains

**Fig. 1:**
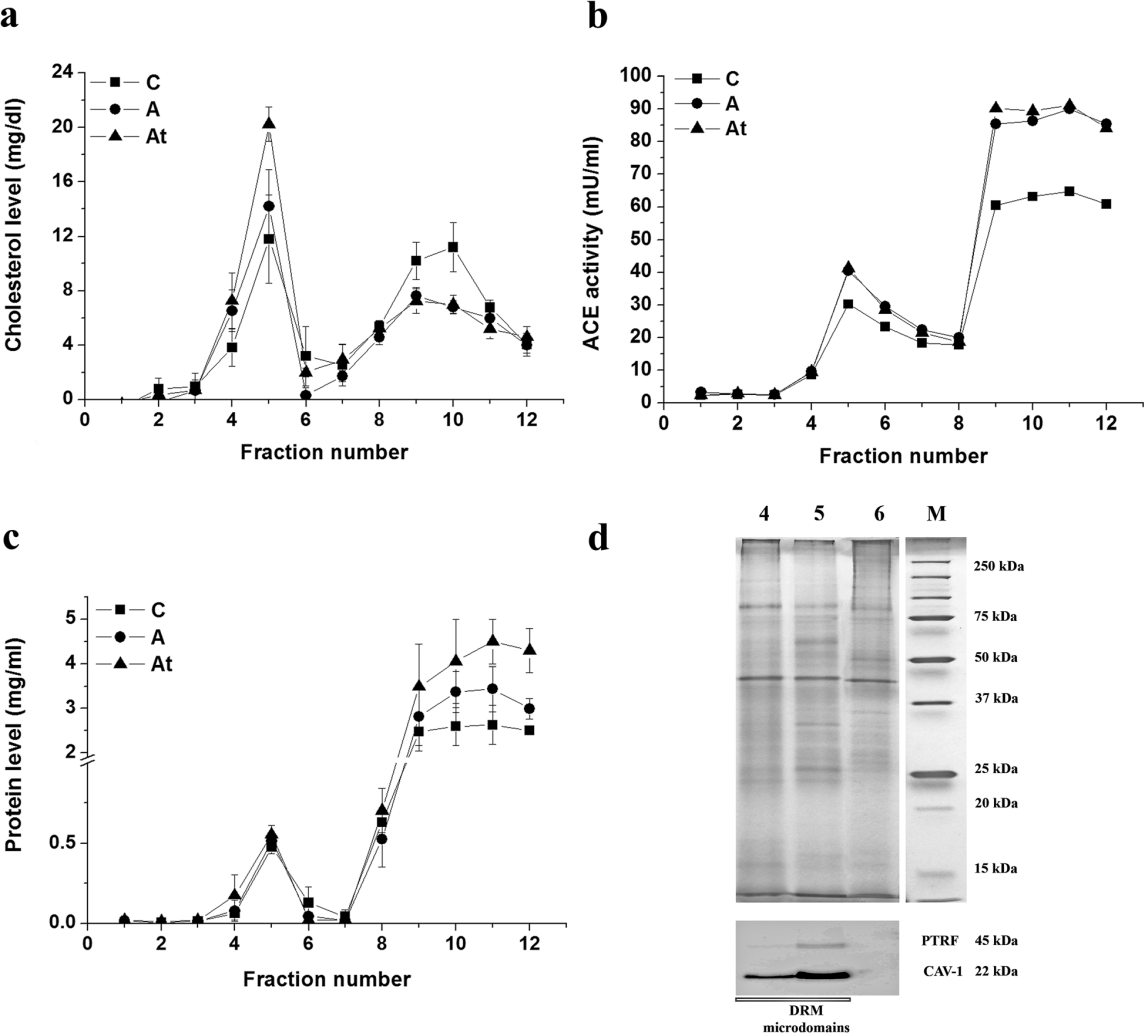
Biochemical parameters and validation of detergent resistant membrane microdomains. Isolated DRM microdomains showing that the 4^th^ and 5^th^ fractions were enriched in cholesterol (**a**), protein (**c**) and presenting high ACE activity (**b**). Representative silver stained 1D SDS-PAGE and Western Blot images of fractions 4 through 6 (**d**). Immunoblot experiments were performed for caveolin-1 and polymerase I and transcript release factor (PTRF) as DRM microdomain protein markers. Fractions 4 and 5 were chosen for further proteomic experiments as DRM microdomains enriched fractions. Molecular weight protein standards (M) are also shown

DRM microdomains were isolated from lung tissue, which has the highest surface of endothelial cells in a body. The biochemical determinations performed on the sucrose gradient fractionated Triton X-100 extract revealed the 4^th^ and the 5^th^ fractions as the right candidates for further proteomic analysis. Namely, both cholesterol and protein levels were increased in these fractions (Fig. 1a, c). In addition, the concentration of cholesterol was higher both in the A and At groups as opposed to the control C (Fig. 1a). The small difference observed between the cholesterol level in the 4^th^ and 5^th^ fractions of DRM isolated from A and At groups proved to be non-statistically significant. To assess that DRM microdomains were mostly of endothelial origin we positively identified ACE protein (P09470 Uniprot access code for angiotensin I converting enzyme), a marker of endothelial cell plasma membrane, through LC-MS/MS experiments (with a Mascot score of 125.91) and measured its activity. Indeed, the fractions enriched in DRM microdomains presented a higher level of ACE activity. Moreover, the ACE activity of the atherosclerotic animal group A was found to be higher than in the control group C suggesting an activation of the endothelial cells under hyperlipidemia stress (Fig. 1b).

In the present study, caveolin-1, polymerase I and transcript release factor (PTRF) together with flotilin 1 [7, 31], glycosylphosphatidylinositol (GPI)-anchored proteins, namely Thy-1 membrane glycoprotein and carboxypeptidase M, accepted as DRM resident proteins [31, 47, 48], were also identified (with Mascot scores > 200) by mass spectrometry. In the present data mining extension of the same experimental model, the caveolin-1 and PTRF were also confirmed by immunological detection (Fig. 1d) to be enriched in fractions 4 and 5, thus validating the DRM isolation. Additionally, mass spectrometry experiments evidenced high identification Mascot scores for caveolin-1 and PTRF (335.21 and 828.94 respectively).

### Shotgun proteomic qualitative and label-free quantitative analysis

**Fig. 2:**
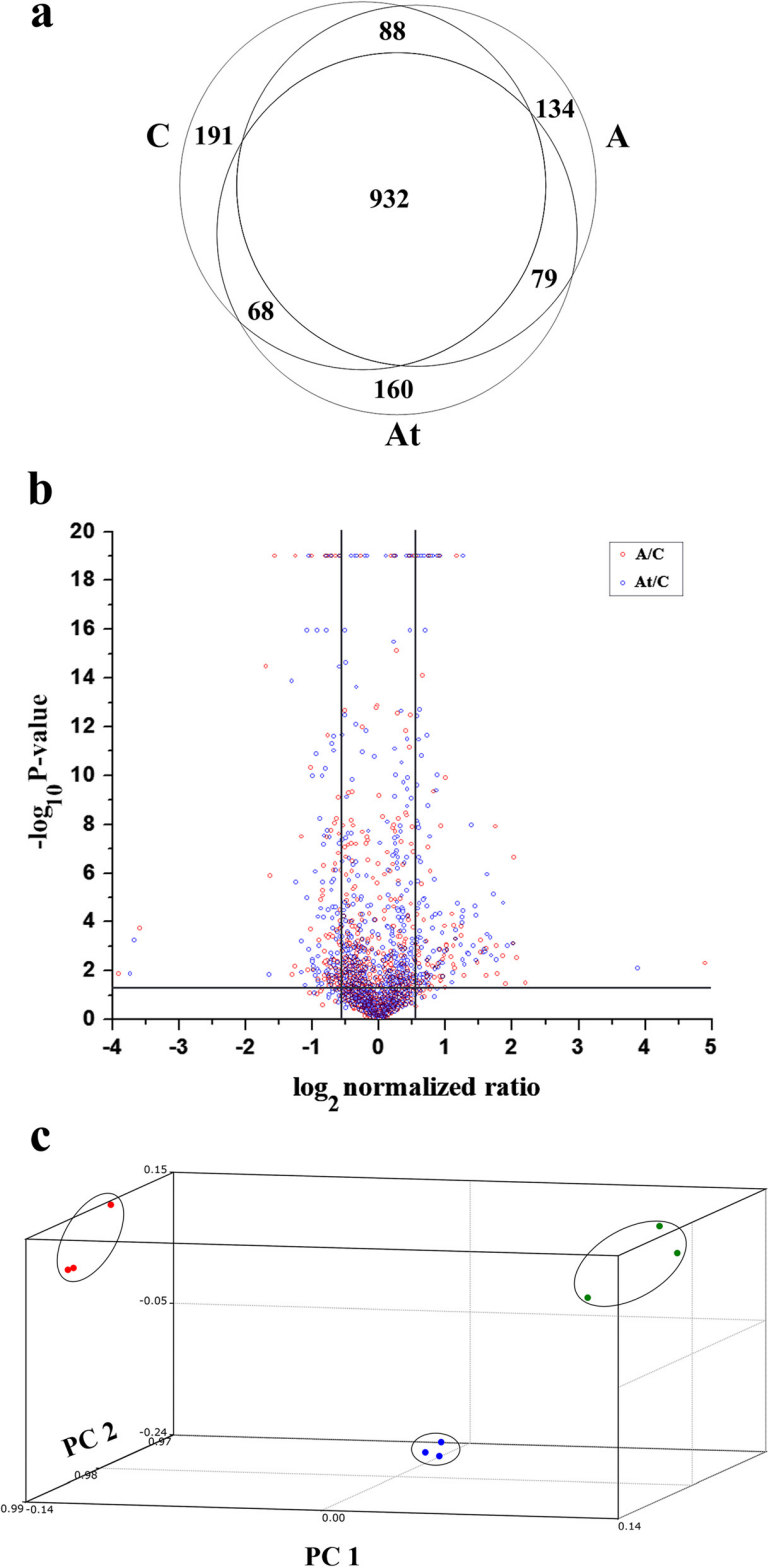
Liquid chromatography – tandem mass spectrometry data. **a** Numerical distribution diagram showing the identified proteins in detergent resistant membrane microdomains in: control group (C: 1279 proteins), ApoE KO mice that received hyperlipidemic diet (A: 1233 proteins) and ApoE KO mice fed hyperlipidemic diet followed by statin treatment (At: 1239 proteins). Commonly identified (932 proteins) as well as uniquely attributed proteins (C: 191 proteins; A: 134 proteins and At: 160 proteins) are depicted. **b** The normalized ratio was plotted against the significance level for the proteins which were altered either in the hyperlipidemic condition (A/C: red circles) or the statin treatment in genetic hyperlipidemic stress (At/C: blue circles) or both of them. The horizontal line represents the minimum significance value threshold (*P* < 0.05), while the vertical lines denote the 1.5 fold alteration cut-off. The purple color signifies the superposition of red and blue circles. **c** Spatial quantitative scattering profile for the biological replicates of each group was performed for the control group (blue dots), ApoE KO mice fed hyperlipidemic diet (green dots) and ApoE KO mice that received hyperlipidemic diet followed by statin treatment (red dots)

The DRM microdomains comparative shotgun proteomic LC-MS experiments revealed a high plethora of resident and membrane-associated proteins involved in molecular interactions and enzymatic functions. High performance nano-liquid chromatography mass spectrometry experiments were conducted for proteome characterization of isolated DRM microdomains. Thus, 1279 proteins were identified in the control (C), 1233 proteins in the atherosclerotic samples (A) and 1239 proteins came from the treated animals (At), using raw file combination for replicate samples (Fig. 2a).

Label free relative quantification procedure revealed a total of 291 differentially expressed proteins located in DRM microdomains of the A and/or At groups, relative to the control group (Additional file 2: Table S1). The chromatographic alignment of the biological groups revealed a nearly perfect overlapping of the base peak chromatograms (shown in Additional file 3: Figure S1), with the lowest calculated alignment score of 0.882, indicative of the fact that the framing process gave rise to high confidence in detecting low variation m/z and retention time values between biological groups.

For the entire experiment we considered only the peptides with unique protein assignments resulting in a total of 1925 proteins (10180 uniquely assigned peptides) passing the selection criteria (see the Methods section). The retained 291 proteins passing the variance and significance relevance criteria were selected from a total of 29993 detected frames and 1925 imported proteins (Fig. 2b). We also chose to select certain proteins for discussion which were in close proximity of the variance and significance thresholds. For quality assessment of experiment input Principal Component Analysis (PCA) was performed within SIEVE 1.4. PCA represents an unsupervised clustering algorithm used to discover and to reduce the dimensionality of a data set, simultaneously retaining the information present in the data [49, 50]. Three variables, the so-called principal components, were generated using the Sieve PCA algorithm (Fig. 2c). The updated, post-filtering 3D representation of the principal component analysis revealed the excellent differentiation of the atherosclerosis (A) and statin treated atherosclerosis (At) vs. control (C) group. The distinct spatial scattering of the two biological conditions against the control shows different quantification profiles of the features detected in the precursor chromatograms and thus a validation of the proteomic alteration affecting DRM microdomains proteins of the genetic induced hyperlipidemic mice (A group). The statin treatment (At group) induced a shift in the quantification pattern as opposed to the wild type (C group), as well.

Gene ontology (slim version) data from Protein Center of the biological replicates revealed that the majority of the identified proteins are in fact of membrane origin (shown in Additional file 4: Figure S2). We have to stress out though that the classification by the different categories is not absolute and may in fact cause often data overlapping of protein function or localization. There are few studies in the literature that describe DRM proteome using the Gene Ontology database [51–53], in particular for the lung isolated DRMs, so insight into the localization, molecular function and biological process of the DRMs’ proteins will be valuable for future studies. Different classes were particularly well represented in the pool of proteins identified against the pre-existent Gene Ontology Slim data protein classification. For example: the cytoplasm and membrane origin proteins in the *Cellular Components*, cell communication, cell organization and biogenesis, metabolic process and regulation of biological process proteins in the *Biological Processes* and protein binding, catalytic activity, metal ion binding, nucleotide binding and transporter activity proteins in the *Molecular Function* appeared to be preferentially modified in hyperlipidemia.

### Analysis of DRMs’ protein expression in actin-dependent signaling pathways

**Fig. 3:**
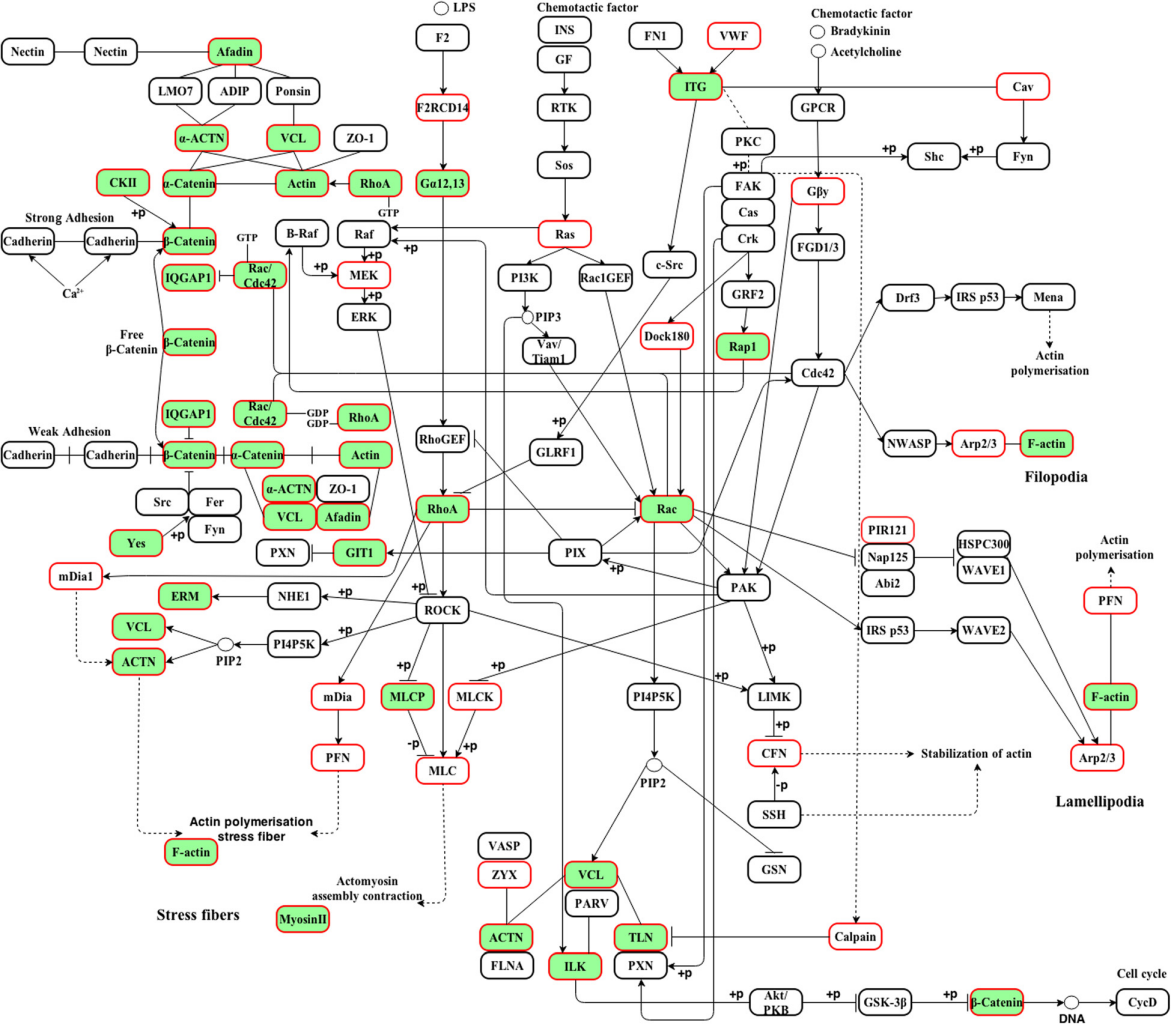
Actin dependent signal transduction pathways of identified and quantified detergent resistant membrane microdomains proteins. The interaction map comprises the currently discussed mass spectrometric identified proteins (red bordered boxes) and differentially expressed proteins (green filled red bordered boxes) and were integrated in the over-represented *Regulation of actin cytoskeleton*, *Focal adhesion* and *Adherens junction* signaling pathways. The complex signaling network represents the combination of *map04810*, *map 04510* and *map 04520* KEGG signaling pathways. Abbreviations for the discussed proteins: Ras: Ras-related protein R-Ras; MEK: mitogen-activated protein kinase kinase 1; F2RCD14: coagulation factor II, CD14 antigen; DOCK180: dedicator of cytokinesis protein; VWF: von Willebrand factor; Cav: caveolin; Gβγ: Guanine nucleotide-binding protein G(I)/G(S)/G(O) subunit gamma-12; Arp2/3: Actin-related protein 2/3 complex subunit 1B and 3; PIR121: Cytoplasmic FMR1-interacting protein 1; PFN: profilin; CFN: Cofilin-1; ZYX: Zyxin; MLC: Myosin regulatory light polypeptide 9; MLCK: Myosin light chain kinase 2; mDia: Protein diaphanous homolog 1; ACTN: actinin: VCL: vinculin; TLN: talin; ITG: integrin; Rap-1: Ras-related protein Rap-1A; Rac: Ras-related C3 botulinum toxin substrate 1; ILK: Integrin-linked protein kinase; MLCP: Serine/threonine-protein phosphatase PP1-beta catalytic subunit; RhoA: Transforming protein RhoA; ERM: ezrin/radixin/moesin; GIT1: ARF GTPase-activating protein GIT1; Yes: Tyrosine-protein kinase Yes; IQGAP1: RasGTPase-activating-like protein IQGAP1; CKII: Casein kinase II subunit alpha; Gα12,13: Guanine nucleotide-binding protein subunit alpha-13

**Fig. 4:**
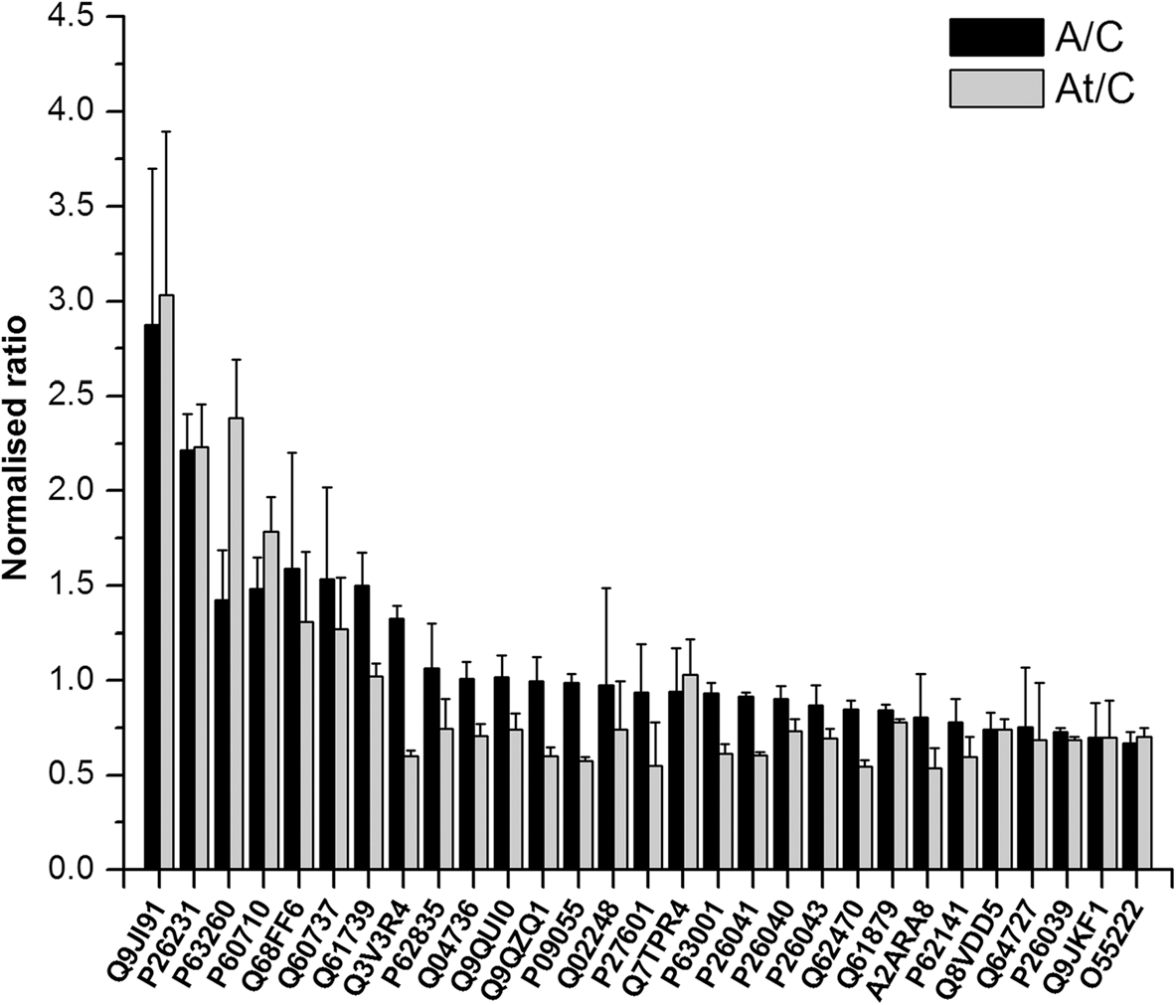
Graphical overview of differentially expressed proteins identified in actin dependent signal transduction pathways. The proteins listed in Table 1 were found to be part of *Regulation of actin cytoskeleton*, *Focal adhesion* and *Adherence junction* over-represented signaling pathways targeted by hyperlipidemia and statin therapy

Hyperlipidemia and statin treatment demonstrated alteration of the DRMs’ protein expression in selective signaling pathways. The LC-MS/MS comparative analysis examined in Protein Center evidenced 13 over-represented statistically significant signaling pathways (Additional file 5: Table S2), (FDR *P-value* < 0.05) and relevant for the cellular processes that revealed various numbers of up- or down-regulated proteins. Among them, proteins from three signaling pathways, namely: *Regulation of actin cytoskeleton* (FDR *P-value* = 8.56E-4), *Focal adhesion* (FDR *P*-value = 9.36E-3) and *Adherens junction* (FDR *P-value* = 2.62E-4) proved to be particularly modified under the hyperlipidemic stress. The extreme interdependency of the three chosen KEGG signaling pathways at the cytoskeleton-membrane-exoplasmatic leaflet of the endothelial cell interface was the main reason for their selection, as it is well evidenced in the very complex Fig. 3. Fluvastatin therapy showed selective outcome on different proteins in these pathways. The 29 proteins found to be involved in the mentioned inter-related membrane cytoskeleton pathways were listed with the annotation parameters in Table 1 and represented in Fig. 4. In the first stage, the present paper will be focused on these three over-represented signaling pathways analysis; the others will follow shortly.

**Table 1:** Catalogue of differentially expressed proteins of DRM microdomains isolated from ApoE KO mice fed hyperlipidemic diet (A) and ApoE KO mice that received hyperlipidemic diet and statin treatment (At) against the control group (C). Table reports the SwissProt/UniProt Accession Number, protein description, molecular weight (MW), Mascot identification score, SIEVE normalized ratios of A over C and of At over C, their respective standard deviations and *p-values*

UNIPROT KEY	DESCRIPTION	MW (kDa)	Mascot score	A/C ratio	A/C std. dev.	A/C *p* value	At/C ratio	At/C std. dev.	At/C *p* value
Q9JI91	Alpha-actinin-2	103.8	353.78	2.875	0.825	1.84E-03	3.030	0.865	1.72E-03
P26231	Catenin alpha-1	100.1	100	2.212	0.193	5.04E-04	2.230	0.226	5.03E-04
P63260	Actin, cytoplasmic 2	41.8	5740.2	1.422	0.265	1.49E-02	2.386	0.305	1.10E-04
P60710	Actin, cytoplasmic 1	41.7	5483.9	1.483	0.163	4.84E-05	1.785	0.184	1.80E-06
Q68FF6	ARF GTPase-activating protein GIT1	85.2	62.49	1.590	0.610	6.57E-02	1.308	0.369	8.19E-02
Q60737	Casein kinase II subunit alpha	36.5	45.1	1.532	0.485	8.67E-02	1.267	0.274	5.76E-02
Q61739	Integrin alpha-6	97.3	122.1	1.499	0.175	1.12E-03	1.022	0.066	2.41E-01
Q3V3R4	Integrin alpha-1	130.7	236.53	1.325	0.065	3.38E-09	0.598	0.0308	3.26E-08
P62835	Ras-related protein Rap-1A	21.0	444.1	1.063	0.235	4.03E-01	0.747	0.156	8.04E-02
Q04736	Tyrosine-protein kinase Yes	92.38	60.6	1.008	0.090	5.49E-01	0.706	0.066	8.97E-05
Q9QUI0	Transforming protein RhoA	21.8	242.8	1.018	0.114	7.23E-01	0.741	0.083	1.42E-02
Q9QZQ1	Afadin	202.0	206.4	0.996	0.129	1.84E-03	0.559	0.051	2.40E-06
P09055	Integrin beta-1	88.2	333.2	0.989	0.048	5.76E-01	0.575	0.021	5.21E-11
Q02248	Catenin beta-1	85.4	2060.4	0.973	0.514	9.08E-01	0.740	0.255	1.97E-01
P27601	Guanine nucleotide-binding protein subunit alpha-13	44.0	108.65	0.938	0.252	6.44E-01	0.550	0.230	3.39E-02
Q7TPR4	Alpha-actinin-1	103.0	1300.8	0.940	0.229	3.63E-02	1.029	0.190	3.50E-02
P63001	Ras-related C3 botulinum toxin substrate 1	21.4	111.49	0.932	0.057	2.94E-01	0.611	0.052	2.37E-06
P26041	Moesin	67.7	1657.9	0.913	0.024	2.01E-02	0.605	0.017	9.9E-20
P26040	Ezrin	69.4	594.85	0.904	0.068	3.22E-03	0.732	0.062	4.39E-04
P26043	Radixin	68.5	691.5	0.871	0.106	3.00E-01	0.694	0.053	3.37E-05
Q62470	Integrin alpha-3	116.7	178.09	0.846	0.049	2.24E-02	0.543	0.0373	5.97E-09
Q61879	Myosin-10	228.9	3486.6	0.843	0.028	2.04E-08	0.778	0.019	9.9E-20
A2ARA8	Integrin alpha-8	117.5	69.83	0.803	0.232	2.51E-01	0.537	0.108	1.11E-02
P62141	Serine/threonine-protein phosphatase PP1-beta catalytic subunit	37.2	61.83	0.779	0.124	5.84E-02	0.598	0.103	1.50E-02
Q8VDD5	Myosin-9	226.2	7387	0.741	0.089	4.55E-03	0.742	0.056	2.71E-03
Q64727	Vinculin	16.6	2596.1	0.755	0.315	2.53E-01	0.685	0.301	1.69E-01
P26039	Talin-1	269.7	3143	0.728	0.022	5.00E-10	0.684	0.020	2.19E-12
Q9JKF1	RasGTPase-activating-like protein IQGAP1	188.7	188.6	0.696	0.186	6.47E-02	0.699	0.193	6.48E-02
O55222	Integrin-linked protein kinase	51.3	330.25	0.668	0.060	9.41E-05	0.704	0.045	1.05E-03

#### *Regulation of actin cytoskeleton* pathway

The Gene Ontology analysis of the 291 differentially expressed proteins of the isolated DRM microdomains demonstrated notable enrichment in membrane and cytoskeleton proteins. The *regulation of actin cytoskeleton* was found as an over-represented KEGG pathway map (Fig. 3), with 21 proteins (including protein species) out of a total of 159, which were identified and differentially expressed in the label free quantification process. While the expression of some proteins was drastically up-regulated by hyperlipidemic stress, others were down-regulated or seemed to be unaffected significantly (Table 1). For example, actin cytoplasmatic 1 and 2 were found over-expressed both in the hyperlipidemic model (with ratios over the control of 1.48 ± 0.16 and 1.42 ± 0.26 respectively) as well as in the treated hyperlipidemic group (with ratios over the control of 1.78 ± 0.18 and 2.38 ± 0.30 respectively). A similar trend was found for alpha actinin 2 and ARF GTP-ase-activating protein GIT1. The alpha-3 and alpha-8 integrins were found significantly under-expressed in the treated hyperlipidemic group, with the hyperlipidemic condition having almost no effect over their expression. Similarly, integrin alpha-1 was found significantly under-expressed in the statin treatment group, but the hyperlipidemic condition up-regulated its expression.

In both A/C and At/C cases, a lower protein expression was revealed also for guanine nucleotide-binding protein subunit alpha-13, integrin beta-1, moesin, radixin, Ras-related C3 botulinum toxin substrate-1, serine/threonine-protein phosphatase PP1 beta catalytic subunit and vinculin, presenting a more pronounced character in the statin treatment group. Ezrin, myosin-10, myosin-9 and transforming protein RhoA were found as well under-expressed (affected especially by the statin treatment). Alpha actinin was also identified in this signaling pathway, although its expression was not significantly altered in either two conditions when compared to control group. Regarding the changes induced by fluvastatin treatment, different results could be distinguished. Comparing the A/C with At/C rations revealed either up- or down-regulation tendency for the majority of the identified proteins. However, the effect is reversed for a few proteins: alpha-actinin-1, transforming protein RhoA and integrin alpha-1 as shown in Fig. 4.

**Fig. 5:**
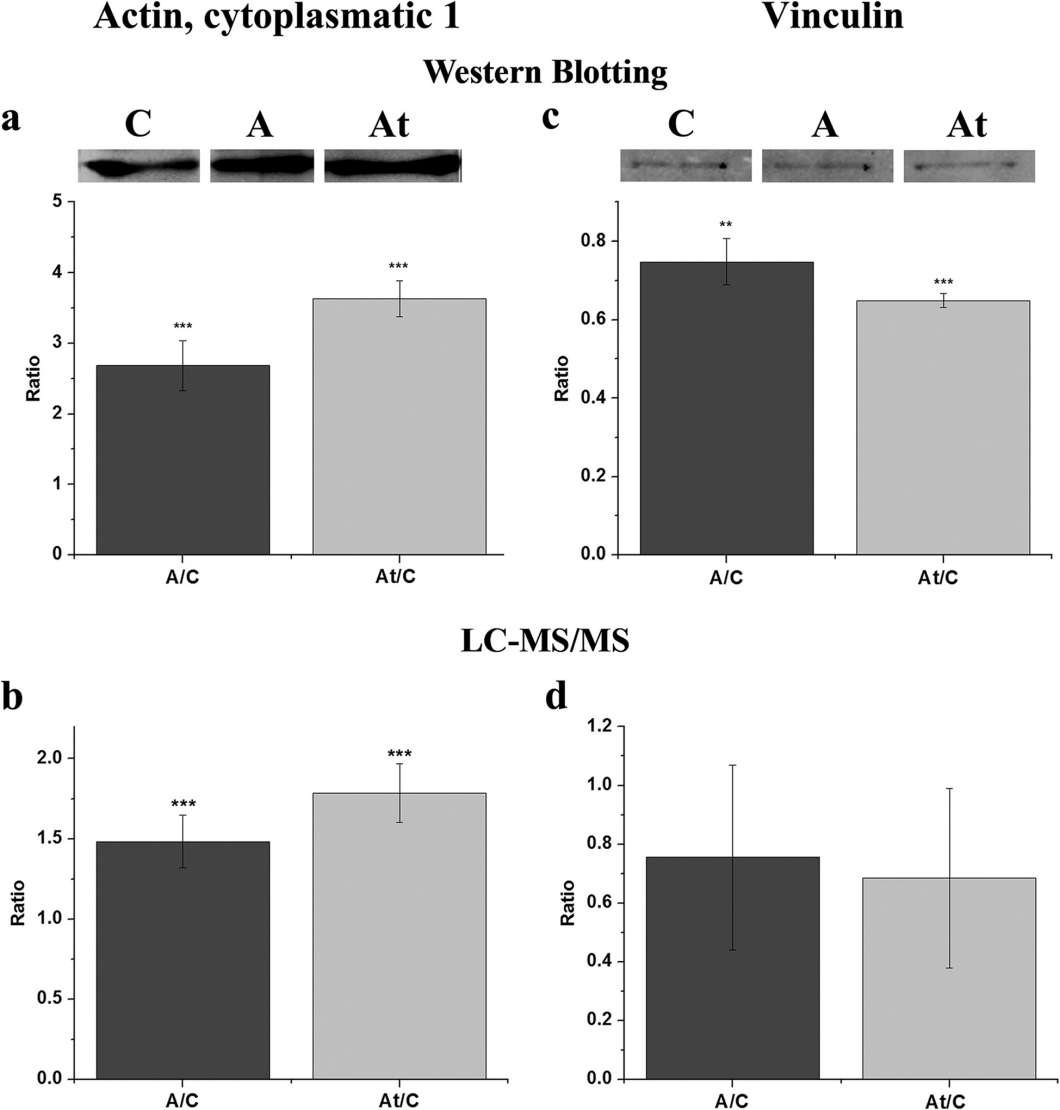
Immunological validation of mass spectrometry data for beta-actin and vinculin. Protein expression of beta-actin (actin cytoplasmatic 1) and vinculin was detected in membrane microdomains enriched fractions by Western Blotting (**a** and **c** respectively), confirming the alteration caused by the hyperlipidemic condition (A) and statin treatment (At) when compared to the control samples (C), detected by LC-MS/MS analysis (**b** and **d** respectively). The Western Blotting experiments were repeated three times. Data are expressed as means ± SD. **p < 0.01, ***p < 0.001

Immunological validation of actin, cytoplasmatic 1 (P60710, also known as beta-actin) and vinculin (Q64727) abundance alteration detected by mass spectrometric analysis was performed using the Western Blotting methodology (Fig. 5). The experiments confirmed with high significance that indeed the hyperlipidemic condition and statin treatment lead to a higher protein expression in the case of beta-actin when compared to the control samples (A/C: 2.683 ± 0.354; At/C: 3.633 ± 0.251) and a lower expression of vinculin (A/C: 0.747 ± 0.05; At/C: 0.649 ± 0.01).

#### *Focal adhesion* proteins

Another KEGG signaling pathway that was found over-represented by the proteins that were differentially expressed in A and At animals was the one that involves *focal adhesion* proteins (Fig. 3). Besides the inherently common proteins involved in the process of regulation of actin cytoskeleton, some other proteins demonstrated altered expression: integrin-linked protein kinase presented a statistically significant lower expression in the hyperlipidemic animal group. A more pronounced under-expression caused by the statin treatment was observed for Ras-related protein Rap-1A and talin-1. Catenin beta-1 was also down-regulated in the At group, although not statistically significant. The hyperlipidemia compensatory effect of statin therapy could be observed for few proteins, three of them being involved in both actin cytoskeleton and focal adhesion regulation pathways: alpha-actinin-1, transforming protein RhoA and integrin alpha-1 besides the ras-related protein Rap-1A (Fig. 4). In total, 17 out of the 145 proteins that map the KEGG pathway were found to be differentially expressed supporting the altered organization of intercellular focal adhesion in hyperlipidemia.

#### *Adherens junction* proteins

The adherent junction KEGG signaling pathway was also over-represented by the statistical Protein Center analysis, with 12 differentially expressed proteins out of 55 possible candidates (Fig. 3); amongst them, tyrosine-protein kinase Yes was unaffected by the hyperlipidemic condition (A), whereas the statin treatment (At) significantly lowered its expression. Catenin alpha-1 was significantly up-regulated both by the hyperlipidemic condition (A) as well as by statin treatment (At). Casein kinase II subunit alpha presented a higher expression in the atherosclerotic animals, while the statin treatment altered its expression, with an A/C ratio reaching almost the control level (Table 1).

Other proteins that are integrated into these three above mentioned signaling pathways that were identified by the present LC/MS-MS experiments, but were not found to be differentially expressed were included as Additional file 6: Supplementary information.

## Discussion

High performance LC-MS proteomic analysis evidently demonstrated that the replicates of the same biological condition discussed in this work, by *Principal component analysis* (PCA), clustered together on the same surfaces of the PCA 3D graphical representation (Fig. 2c). At the same time, the three experimental conditions (C, A, and At) revealed a distant partitioning from one another, corroborated with the different proteomic composition of the three experimental groups. Thus, the PCA applied test showed that the differences found between the three sample groups were consistent, underlying a biological significance.

Since hyperlipidemia is the main risk factor in the development of atherosclerosis and our previous data [7] demonstrated the specific induced changes in the proteomic composition of membrane microdomains, we extended the proteomic analysis. LC-MS/MS technology allowed the identification with high confidence (high ion scores, large protein identification coverage) of 29 proteins associated with DRM microdomains isolated from control (C), atherosclerotic ApoE deficient (A) and statin treated ApoE deficient mice (At). These proteins resulted to be associated with three key signaling pathways that proved to be targeted by the hyperlipidemic stress. In the present study, we focused on the interaction of DRM proteins with the cytoskeleton elements resulting in regulating membrane microdomains-associated signaling events since the actin-dependent signal transduction pathways (*Actin cytoskeleton, Focal adhesion proteins* and *Adherens junction proteins*) are over represented in our experimental condition. It is evident that hyperlipidemia induced profound changes in the expression (up- or down-regulated) of 291 proteins with different cellular location and function and 29 of them are closely related to the DRM-cytoskeleton events. The results are not surprising since the repetitive events of polymerization/depolymerization of the monomer units of actin is the main source of energy and movement for multiple biological functions that take place in the cells and at the plasma-membrane level [54]. Endothelial-derived foam cells showed a modified pattern of actin and vinculin localization [55]. From the current mass spectrometric study, we can also evidence a cytoskeleton alteration. We observed that the statin treatment doesn’t diminish the hyerlipidemic stress up to the basal level, but determines an even more pronounced effect on the actin and vinculin mass spectrometric abundance levels (Fig. 5), (a higher normalised ratio of At/C than A/C in the case of actin and a smaller normalised ratio of At/C than A/C in the case of vinculin). The exposure of endothelial cells in culture to oxLDL (as atherogenic risk factor) also induced low plasma membrane expression of the lipid raft marker GM1 [56] and internalization of endothelial caveolin 1 [57]. Previous studies demonstrated that inhibition of actin filament polymerization interferes with vasodilatory signaling in human coronary arterioles and the pulmonary circulation, which may suggest a crucial role for endothelial cytoskeleton integrity in modulating endothelial-dependent vasodilator signal transduction [58–60]. Although statins are known to reduce lung inflammation through tetraspanins modulation [61], there is plenty of published evidence that statin treatment may have side effects by inducing mild to severe myopathy, increased risk of diabetes and abnormalities in liver enzyme tests in patients receiving this drug [62]. In this context, our results regarding the significant modulation in the expression of actin cytoskeleton proteins are highly informative for pinpointing several altered protein molecules that could provide the basis for future functional studies. Previously published immunohistochemistry data support our results that alteration in the expression and distribution of the cytoskeleton proteins, for example actin, ezrin and cytokeratin-7, is present when normal and pathological membrane microdomains were analyzed. Thus, DRMs from intrauterine growth restriction (IUGR) and preeclampsia (PE) pathologies showed clear different features when compared to rafts from normal placenta, underlining the consistency of the mass spectrometry methodology used to reveal the stress induced proteomic and signal transduction changes [63].

Some studies [64, 65] suggest a role of DRM microdomains in regulating cytoskeleton-dependent processes, including proliferation, trafficking, signaling, migration and polarization. A malfunction or deregulation of DRMs can interfere with the cytoskeleton interaction, blocking essential cellular functions. In the very well established model of immunological synapse, bidirectional interaction takes place between membrane microdomains and the actin cytoskeleton, in which signaling molecules localized in DRM microdomains are partially responsible for the reorganization of the actin cytoskeleton, while in turn this is required for coalescing of the resident DRM (*lipid rafts*) proteins necessary for the immunological synapse. Additionally, raft-cytoskeleton interaction has been demonstrated to take place in other cell types, as it is the case for migration of polarized cells, where DRMs act as organizational microdomains for membrane receptors, signaling molecules and regulators of actin cytoskeleton.

It has been well proven that hypercholesterolemia is associated with elevated levels of oxLDL [66–69]. OxLDL can promote atherosclerosis development by inducing the recruitment of platelet-activating factor receptor and CD36 in detergent resistant membranes [70]. Another study has demonstrated that oxLDL can induce the depletion of cholesterol from endothelial caveolae (a vesicular-type subset of detergent resistant membrane microdomains) which promotes disruption of these cholesterol rich microdomains affecting associated processes and thus endothelial dysfunction [71]. These observations are consistent with the fact that hypercholesterolemia can result in the depletion of plasma membrane cholesterol, evidenced in a report on the currently discussed diet-induced hypercholesterolemic apoE-deficient mice [72]. Different studies have demonstrated that oxLDL can induce rapid polymerization of actin and in turn, the formation of filamentous actin and actin stress fibers in vascular endothelial cells [73] and monocytes [74] or macrophages [75]. The authors concluded that oxLDL induces the formation of actin stress fibers through the activation of RhoA/Rho kinase pathway, observation confirmed by another study performed on HUVECs [76].

Transforming protein RhoA and Ras-related C3 botulinum toxin substrate 1 (Rac1) are members of the Rho family of small GTP-binding proteins. These proteins, identified and quantified in our study as well, play key roles in the organization of the cytoskeleton and also in its coupling to the plasma membrane [77, 78]. It has been demonstrated that the activation of Rac type proteins induces membrane ruffling, while Rho, once activated, determines the formation of stress fibers and also regulates cell polarization and migration, trafficking or proliferation and it is also implicated in the formation of cell-extracellular matrix focal adhesions [79, 80]. Other studies have shown that these proteins, once activated, are translocated from cytoplasm, via integrin signaling [81, 82] to plasma membrane and concentrate in DRM microdomains [83, 84]. Integrin signaling is important as well for the control of Rho activity and can be modulated through mechanical changes in the cytoskeleton [85]. Also, it has been demonstrated that lipid rafts are mediating growth cone guidance in the angiogenetic process, through localized assembly of receptor-ligand interactions, the afterwards cytoskeletal rearrangement and local protein synthesis, including Src family kinases and Rho GTPases [86].

We do acknowledge the fact that the present proteomic findings may be attributed to the cellular heterogeneity in correspondence with the DRMs’ isolation source. However, the high level of ACE activity (specific marker for the luminal surface of endothelium) measured in the DRM enriched fractions (Fig. 1b) corroborated with the extremely large surface of the pulmonary endothelium provide us the basis to believe that the endothelial cells were the major source of DRM extraction. Nevertheless, recent publications [87] claimed the specific localization of ACE2 on the lung epithelial cells. Indeed the procedure used to isolate DRMs does not exclude the presence of epithelial contribution to the protein profile of DRMs. The specific enzymatic activity measurement based on Hip-His-Leu substrate, and the mass spectrometric identification of the angiotensin I converting enzyme (P09470 Uniprot access code) positively support our statement.

## Conclusions

Taken together, these data demonstrate clearly that DRM microdomains are indeed essential biological structures that preserve the integrated macromolecular components involved in the key pathways that maintain the cellular homeostasis and could be deregulated under stress factors, including hyperlipidemia. The differential proteomic analysis proposed in this experimental strategy aimed to identify the biological processes and key proteins located at membrane level. The research strategy was based on the concept that DRM microdomains are the main platforms of protein-protein and protein-lipid interactions. These connections fulfill numerous biological functions governed by controlled energy production and specific signaling pathways that are maintained after the drastic mechanical procedure used during the preparation. The study, objectively and reliably, revealed a panel of proteins of interest in hyperlipidemia that may easily generate new downstream research applications. The complex network of interactions and dependency between the proteins involved in these dynamic pathways, evidenced by the mass spectrometry analysis, may be used to design experiments in which to study the different protein-protein interactions under hyperlipidemic stress.

Only recently, the most complete and up to date lipid raft proteome database has been published [88]. It includes mammalian lipid raft associated proteins reported by using various biochemical isolation methods and high throughput analysis such as mass spectrometry studies. Already including published results of our group [7], this paper aims also to add to the present collection a comprehensive list of DRM proteins affected by the hyperlipidemic condition and statin treatment to further enhance the current knowledge on these extraordinary structural and functional molecular platforms.

## Additional files

Additional file 1:
**Mass spectrometry method.** (DOCX 21 kb) Additional file 2: Table S1. Mass spectrometry data of differentially abundant proteins extracted from Protein Center databases. (XLSX 66 kb) Additional file 3: Figure S1. Base peak chromatographic alignment. Representative biological replicates of the three groups are shown: C - control group (blue base peak chromatograms), A - ApoE KO mice fed hyperlipidemic diet (green base peak chromatograms) and At - ApoE KO mice that received hyperlipidemic diet and statin treatment (red base peak chromatograms). (DOCX 277 kb) Additional file 4: Figure S2. Gene ontology details of detergent resistant membrane microdomains proteins. Data distribution based on: Cellular Component (a), Biological Process (b) and Molecular Function (c). Data expressed as mean values ± SD, following the analysis of the biological replicates. (DOCX 654 kb) Additional file 5: Table S2. (XLSX 11 kb) Additional file 6: Supplementary information. (DOCX 10 kb)
