# Supplementary material for: Alteration of actin dependent signaling pathways associated with membrane microdomains in hyperlipidemia
Source: Proteome Sci. 2015 Dec 1;13:30. doi: 10.1186/s12953-015-0087-0 (PMC4666118; doi:10.1186/s12953-015-0087-0)
Supplement: Additional file 1: — Mass spectrometry method. (DOCX 21 kb) [file 12953_2015_87_MOESM1_ESM.docx]

**Additional file 1:**

**Mass spectrometry method**

Creator: LTQ VELOS

MS Run Time (min): 70.00

Sequence override of method parameters not enabled.

Divert Valve: not used during run

Contact Closure: not used during run

Syringe Pump: not used during run

MS Detector Settings:

Real-time modifications to method disabled

Stepped collision energy not enabled

Additional Microscans:

MS2 0 0

MS3 0 0

MS4 0 0

MS5 0 0

MS6 0 0

MS7 0 0

MS8 0 0

MS9 0 0

MS10 0 0

Segment 1 Information

Duration (min): 70.00

Number of Scan Events: 7

Tune Method: nanotune_peptide

Scan Event Details:

1: FTMS + p norm res=60000 o(300.0-2000.0)

CV = 0.0V

2: ITMS + c norm Dep MS/MS Most intense ion from (1)

Activation Type: CID

Min. Signal Required: 1000.0

Isolation Width: 2.00

Normalized Coll. Energy: 35.0

Default Charge State: 7

Activation Q: 0.250

Activation Time: 10.000

CV = 0.0V

3: ITMS + c norm Dep MS/MS 2nd most intense ion from (1)

Activation Type: CID

Min. Signal Required: 1000.0

Isolation Width: 2.00

Normalized Coll. Energy: 35.0

Default Charge State: 7

Activation Q: 0.250

Activation Time: 10.000

CV = 0.0V

4: ITMS + c norm Dep MS/MS 3rd most intense ion from (1)

Activation Type: CID

Min. Signal Required: 1000.0

Isolation Width: 2.00

Normalized Coll. Energy: 35.0

Default Charge State: 7

Activation Q: 0.250

Activation Time: 10.000

CV = 0.0V

5: ITMS + c norm Dep MS/MS 4th most intense ion from (1)

Activation Type: CID

Min. Signal Required: 1000.0

Isolation Width: 2.00

Normalized Coll. Energy: 35.0

Default Charge State: 7

Activation Q: 0.250

Activation Time: 10.000

CV = 0.0V

6: ITMS + c norm Dep MS/MS 5th most intense ion from (1)

Activation Type: CID

Min. Signal Required: 1000.0

Isolation Width: 2.00

Normalized Coll. Energy: 35.0

Default Charge State: 7

Activation Q: 0.250

Activation Time: 10.000

CV = 0.0V

7: ITMS + c norm Dep MS/MS 6th most intense ion from (1)

Activation Type: CID

Min. Signal Required: 1000.0

Isolation Width: 2.00

Normalized Coll. Energy: 35.0

Default Charge State: 7

Activation Q: 0.250

Activation Time: 10.000

CV = 0.0V

Lock Masses:

Pos List Name: PDMS_371

Source: API Source

Mass List: 371.101240

Neg List Name: N/A

Source: API Source

Mass List: (none)

Data Dependent Settings:

Use separate polarity settings disabled

Parent Mass List: (none)

Reject Mass List:

-300.3008 301.1440 302.0176 302.3075 302.9167

303.0164 303.1452 303.2340 304.0122 304.2492

305.2497 305.9501 306.9321 307.2426 308.0227

308.2604 309.1533 309.2045 311.0545 311.1684

312.0594 313.2357 314.0836 314.1432 317.2861

317.7017 317.9909 318.0492 318.1991 319.2230

320.2590 321.0626 321.2403 321.9226 322.2753

322.9781 323.1528 323.9996 324.1637 324.9948

325.0703 326.2013 326.9989 327.0328 327.0791

328.1596 328.1970 329.0065 329.9503 330.0604

330.2654 331.0041 331.0588 331.1358 331.9831

333.0079 334.2536 334.3122 335.2822 336.2526

337.0356 338.8934 338.9488 339.1272 339.6966

339.9721 340.1404 340.9692 341.0169 342.0179

342.0446 342.1744 343.2984 344.2277 344.3179

345.3180 345.9227 346.0319 346.0821 346.8826

347.1155 347.9608 348.9568 349.0180 350.1791

352.3066 352.9002 353.9782 355.0718 356.0725

356.1909 356.2319 356.3903 357.0714 358.2521

358.3355 359.0301 360.0323 362.8543 363.0880

363.9331 364.0918 364.8539 364.9300 365.1097

365.9941 368.1888 369.1245 370.0922 370.2063

370.9618 371.1029 371.3185 372.1037 373.1034

374.1042 375.1108 375.2792 376.2608 377.2724

378.2595 379.3078 379.9979 380.1020 380.3376

380.9739 384.9604 385.9180 387.9541 388.1381

388.2557 389.2250 389.3140 389.7234 389.9491

390.2306 390.5489 390.7313 390.8846 391.0209

391.0809 391.2309 391.2861 391.7317 391.9133

392.0174 392.2884 392.9465 393.2974 394.9196

394.9883 396.3337 397.3373 398.2259 398.7291

399.9014 400.9374 401.1484 401.9362 402.3590

403.9284 404.9406 405.3026 405.9211 406.1528

406.9195 406.9977 407.8848 407.9947 410.8914

412.9306 413.2670 413.8806 415.8746 416.9100

417.1326 417.9112 418.9144 419.3178 422.9725

423.9679 424.3659 424.8993 425.9041 426.8711

427.3159 428.3752 428.9035 429.0910 429.2385

430.0914 430.3906 430.9154 431.0896 431.8615

432.0900 432.8915 433.8861 434.3276 436.8922

437.9798 438.8971 438.9730 440.3603 440.8708

441.3644 441.9257 442.9322 443.1415 444.8755

445.1222 445.2676 445.9661 446.1231 446.9273

446.9990 447.1211 448.1243 448.2783 448.9883

449.1235 449.3233 449.8720 450.9503 451.9096

452.8671 453.9553 454.9094 455.9173 455.9881

456.9033 457.9016 458.9398 459.1751 459.9765

460.1772 460.9427 461.9396 462.1484 462.2940

462.9691 463.1495 464.1468 464.9590 465.1468

466.9249 467.1029 467.8872 468.3954 468.8485

469.9301 470.9201 471.3429 471.8432 471.9676

472.9187 473.9165 474.9093 475.8658 475.9516

476.2008 477.2019 477.9172 478.1994 478.9131

479.9569 480.6108 480.9484 481.9500 482.8594

483.9403 484.3871 484.8146 484.9359 485.3916

485.9019 486.8990 487.9459 488.9066 490.8861

491.8447 492.8863 493.3559 493.8934 494.8961

495.9313 496.4264 496.9291 497.0184 497.9306

498.0175 498.8301 499.0312 499.9082 500.8283

500.9047 503.1096 503.9209 504.1111 505.1067

506.9303 507.8817 508.8759 509.8650 510.9116

510.9642 511.9054 512.4218 512.9078 512.9947

513.9060 513.9914 514.9188 514.9818 515.3693

518.8954 519.1413 519.2239 519.8976 520.1420

520.9835 521.1400 521.9813 522.1427 522.9059

526.2641 526.5980 526.8727 526.9349 527.8832

528.4137 528.8766 528.9705 529.2632 529.4197

529.8909 529.9658 530.8931 532.8819 533.1945

534.1956 534.9199 535.3274 535.8721 536.1681

536.3286 536.8643 536.9577 537.1683 537.9517

538.1668 538.8840 539.1675 539.9480 540.1649

540.4526 542.9172 542.9854 543.8613 544.8418

544.9404 545.9372 546.8694 547.8730 549.8561

550.2207 550.8966 551.2212 551.8445 552.2183

552.9353 553.9279 554.8570 555.9193 556.4505

559.3956 560.8745 561.8813 562.8386 565.9709

566.8978 567.8288 568.9029 569.8978 570.8744

571.8899 572.4397 572.8714 573.4462 573.9561

574.9567 576.8462 577.1313 580.8673 581.9490

582.9253 584.4789 584.8175 585.3190 587.8521

587.9988 588.8468 589.9311 590.9295 591.9310

593.1614 594.1627 595.1629 595.9922 596.8425

597.9167 598.9116 603.9304 604.8274 605.9057

606.9011 607.2133 607.9045 610.1875 611.1883

611.8506 611.9661 612.1856 612.9671 613.1857

613.8947 614.8900 615.8918 616.4663 617.4737

618.8332 619.9127 620.8011 620.9047 621.8779

622.8742 623.8768 624.2392 625.2409 626.2393

627.6488 627.9828 628.3157 628.6513 628.7810

629.8670 630.8628 631.8566 634.3910 634.8060

636.8694 643.8610 644.8456 645.8405 646.8322

648.4120 649.4127 653.8600 654.3546 658.8855

660.4932 661.5011 663.8691 664.9422 665.9375

666.8702 667.1810 668.1805 669.1793 672.9396

673.8232 673.9339 673.9841 674.3345 674.8599

682.8416 684.2067 685.2072 686.2049 687.2045

687.8259 688.9175 689.9021 690.8277 694.8378

695.9257 696.8487 698.8153 704.5197 705.5250

706.7958 711.9069 712.8166 719.8899 725.9126

726.9031 727.8755 728.8401 729.3426 729.8372

735.8624 741.1956 741.8843 742.8827 743.8528

748.5464 749.8716 750.8668 751.8225 752.3159

752.8181 757.8588 758.2256 758.8553 759.2262

760.2240 765.8413 766.8382 777.8347 778.3365

778.8388 779.4510 780.4561 781.4558 781.8130

785.8442 786.3469 786.8397 787.3496 788.9056

789.3953 789.9052 792.5728 792.9004 793.8126

794.9118 795.4478 795.9118 796.8185 797.3356

801.4371 802.9026 804.8021 807.8184 810.8863

811.8833 812.7840 816.8117 818.8194 823.7907

834.7909 847.8803 855.8643 863.8501 864.8558

871.8369 874.8572 877.8566 878.8578 887.8092

940.9583 941.4641 941.9631

Neutral Loss Mass List: (none)

Product Mass List: (none)

Neutral loss in top: 3

Product in top: 3

Most intense if no parent masses found not enabled

Add/subtract mass not enabled

FT master scan preview mode enabled

Charge state screening enabled

Charge state dependent ETD time not enabled

Monoisotopic precursor selection enabled

Non-peptide monoisotopic recognition not enabled

Charge state rejection enabled

Unassigned charge states : rejected

Charge state 1 : not rejected

Charge state 2 : not rejected

Charge state 3 : not rejected

Charge states 4+ : not rejected

Chromatography mode is disabled

Global Data Dependent Settings:

Predict ion injection time enabled

Use global parent and reject mass lists not enabled

Exclude parent mass from data dependent selection not enabled

Exclusion mass width relative to mass

Exclusion mass width relative to low (ppm): 7.00

Exclusion mass width relative to high (ppm): 7.00

Parent mass width by mass

Parent mass width low: 0.5000

Parent mass width high: 0.5000

Reject mass width relative to mass

Reject mass width relative to low (ppm): 7.00

Reject mass width relative to high (ppm): 7.00

Zoom/UltraZoom scan mass width by mass

Zoom/UltraZoom scan mass width low: 5.00

Zoom/UltraZoom scan mass width high: 5.00

FT SIM scan mass width low: 5.00

FT SIM scan mass width high: 5.00

Neutral Loss candidates processed by decreasing intensity

Neutral Loss mass width by mass

Neutral Loss mass width low: 0.5000

Neutral Loss mass width high: 0.5000

Product candidates processed by decreasing intensity

Product mass width by mass

Product mass width low: 0.5000

Product mass width high: 0.5000

MS mass range: 0.00-1000000.00

MSn mass range by mass

MSn mass range: 0.00-1000000.00

Use m/z values as masses not enabled

Analog UV data dep. not enabled

Dynamic exclusion enabled

Repeat Count: 1

Repeat Duration: 30.00

Exclusion List Size: 500

Exclusion Duration: 90.00

Exclusion mass width relative to mass

Exclusion mass width relative to low (ppm): 7.00

Exclusion mass width relative to high (ppm): 7.00

Expiration: disabled

Isotopic data dependence not enabled

Mass Tags data dependence not enabled

Custom Data Dependent Settings:

Not enabled

**Liquid chromatography method**

Program for Dionex Chromatography MS Link

ColumnOven.TempCtrl = On

ColumnOven.Temperature.Nominal =35.0 [°C]

ColumnOven.Temperature.LowerLimit =20.0 [°C]

ColumnOven.Temperature.UpperLimit =40.0 [°C]

EquilibrationTime = 0.5 [min]

ColumnOven.ReadyTempDelta = 1.0 [°C]

Sampler.TempCtrl = On

Sampler.Temperature.Nominal = 5.0 [°C]

Sampler.Temperature.LowerLimit =4.0 [°C]

Sampler.Temperature.UpperLimit =20.0 [°C]

Sampler.ReadyTempDelta = None

LoadingPump.Pressure.LowerLimit =0 [bar]

LoadingPump.Pressure.UpperLimit =400 [bar]

LoadingPump.MaximumFlowRampDown =5 [µl/min²]

LoadingPump.MaximumFlowRampUp =5 [µl/min²]

LoadingPump.%A.Equate = "%A"

LoadingPump.%B.Equate = "%B"

%C.Equate = "%C"

NC_Pump.Pressure.LowerLimit = 50 [bar]

NC_Pump.Pressure.UpperLimit = 500 [bar]

NC_Pump.MaximumFlowRampDown = 0.300 [µl/min²]

NC_Pump.MaximumFlowRampUp = 0.300 [µl/min²]

NC_Pump.%A.Equate = "%A"

NC_Pump.%B.Equate = "%B"

DrawSpeed = 200 [nl/s]

DrawDelay = 5000 [ms]

DispSpeed = 2000 [nl/s]

DispenseDelay = 2000 [ms]

WasteSpeed = 4000 [nl/s]

WashSpeed = 4000 [nl/s]

LoopWashFactor = 2.000

SampleHeight = 4.000 [mm]

PunctureDepth = 8.000 [mm]

WashVolume = 75.000 [µl]

RinseBetweenReinjections = Yes

LowDispersionMode = Off

InjectMode = FullLoop

FlushVolume = 5.000 [µl]

FlushVolume2 = 3.000 [µl]

LoopOverfill = 2.000

LoadingPump_Pressure.Step = 0.01 [s]

LoadingPump_Pressure.Average =Off

NC_Pump_Pressure.Step = 0.01 [s]

NC_Pump_Pressure.Average = Off

LoadingPump.Flow = 5.000 [µl/min]

LoadingPump.%B = 0.0 [%]

%C = 0.0 [%]

LoadingPump.Curve = 5

ValveLeft = 1_2

0.000 Wait LoadingPump.Ready and NC_Pump.Ready and ColumnOven.Ready and Sampler.Ready and PumpModule.Ready

;Chromeleon sets this property to signal to Xcalibur that it is ready to start a run.

ReadyToRun = 1

;Xcalibur sets this property to start the run or injection.

Wait StartRun

NC_Pump.Flow = 0.300 [µl/min]

NC_Pump.%B = 2.0 [%]

Wait LoadingPump.Ready and NC_Pump.Ready and ColumnOven.Ready and Sampler.Ready and PumpModule.Ready

Inject

LoadingPump_Pressure.AcqOn

NC_Pump_Pressure.AcqOn

NC_Pump_Press_RightBlk.AcqOn

NC_Pump_Press_LeftBlk.AcqOn

;Chromeleon sets this property to signal the injection to Xcalibur.

InjectResponse = 1

;Depending on your system configuration it might be necessary to manually insert

;a "Relay" command below in order to send the start signal to the MS.

;Typical syntaxes:

;Pump_Relay_1.Closed Duration =2.00

;UM3PUMP_Relay1.On Duration = 2.00

;Pump_Relay_1.Closed Duration =2.00

;UM3PUMP_Relay1.On Duration = 2.00

;Pump_Relay_1.Closed Duration =2.00

;UM3PUMP_Relay1.On Duration = 2.00

;Pump_Relay_1.Closed Duration =2.00

;UM3PUMP_Relay1.On Duration = 2.00

NC_Pump.Flow = 0.300 [µl/min]

NC_Pump.%B = 2.0 [%]

3.000 ValveLeft = 6_1

NC_Pump.Flow = 0.300 [µl/min]

NC_Pump.%B = 2.0 [%]

55.000 NC_Pump.Flow = 0.300 [µl/min]

NC_Pump.%B = 28.0 [%]

55.100 NC_Pump.Flow = 0.300 [µl/min]

NC_Pump.%B = 90.0 [%]

59.000 NC_Pump.Flow = 0.300 [µl/min]

NC_Pump.%B = 90.0 [%]

59.100 NC_Pump.Flow = 0.300 [µl/min]

NC_Pump.%B = 2.0 [%]

64.000 ValveLeft = 1_2

70.000 NC_Pump.Flow = 0.300 [µl/min]

NC_Pump.%B = 2.0 [%]

LoadingPump_Pressure.AcqOff

NC_Pump_Press_RightBlk.AcqOff

NC_Pump_Press_LeftBlk.AcqOff

NC_Pump_Pressure.AcqOff

InjectResponse = 0

End
