# Supplementary material for: Alteration of actin dependent signaling pathways associated with membrane microdomains in hyperlipidemia
Source: Proteome Sci. 2015 Dec 1;13:30. doi: 10.1186/s12953-015-0087-0 (PMC4666118; doi:10.1186/s12953-015-0087-0)
Supplement: Additional file 3: Figure S1. — Base peak chromatographic alignment. Representative biological replicates of the three groups are shown: C - control group (blue base peak chromatograms), A - ApoE KO mice fed hyperlipidemic diet (green base peak chromatograms) and At - ApoE KO mice that received hyperlipidemic diet and statin treatment (red base peak chromatograms). (DOCX 277 kb) [file 12953_2015_87_MOESM3_ESM.docx]

Additional file 3: Figure S1


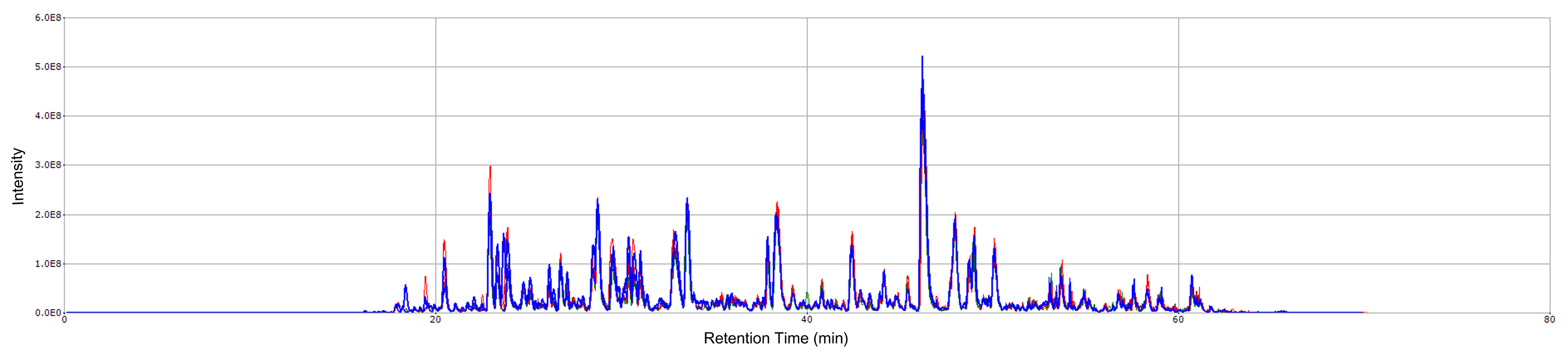


Figure caption:

Base peak chromatographic alignment. Representative biological replicates of the three groups are shown: C - control group (blue base peak chromatograms), A - ApoE KO mice fed hyperlipidemic diet (green base peak chromatograms) and At - ApoE KO mice that received hyperlipidemic diet and statin treatment (red base peak chromatograms)
