# Supplementary material for: Alteration of actin dependent signaling pathways associated with membrane microdomains in hyperlipidemia
Source: Proteome Sci. 2015 Dec 1;13:30. doi: 10.1186/s12953-015-0087-0 (PMC4666118; doi:10.1186/s12953-015-0087-0)
Supplement: Additional file 4: Figure S2. — Gene ontology details of detergent resistant membrane microdomains proteins. Data distribution based on: Cellular Component (a), Biological Process (b) and Molecular Function (c). Data expressed as mean values ± SD, following the analysis of the biological replicates. (DOCX 654 kb) [file 12953_2015_87_MOESM4_ESM.docx]

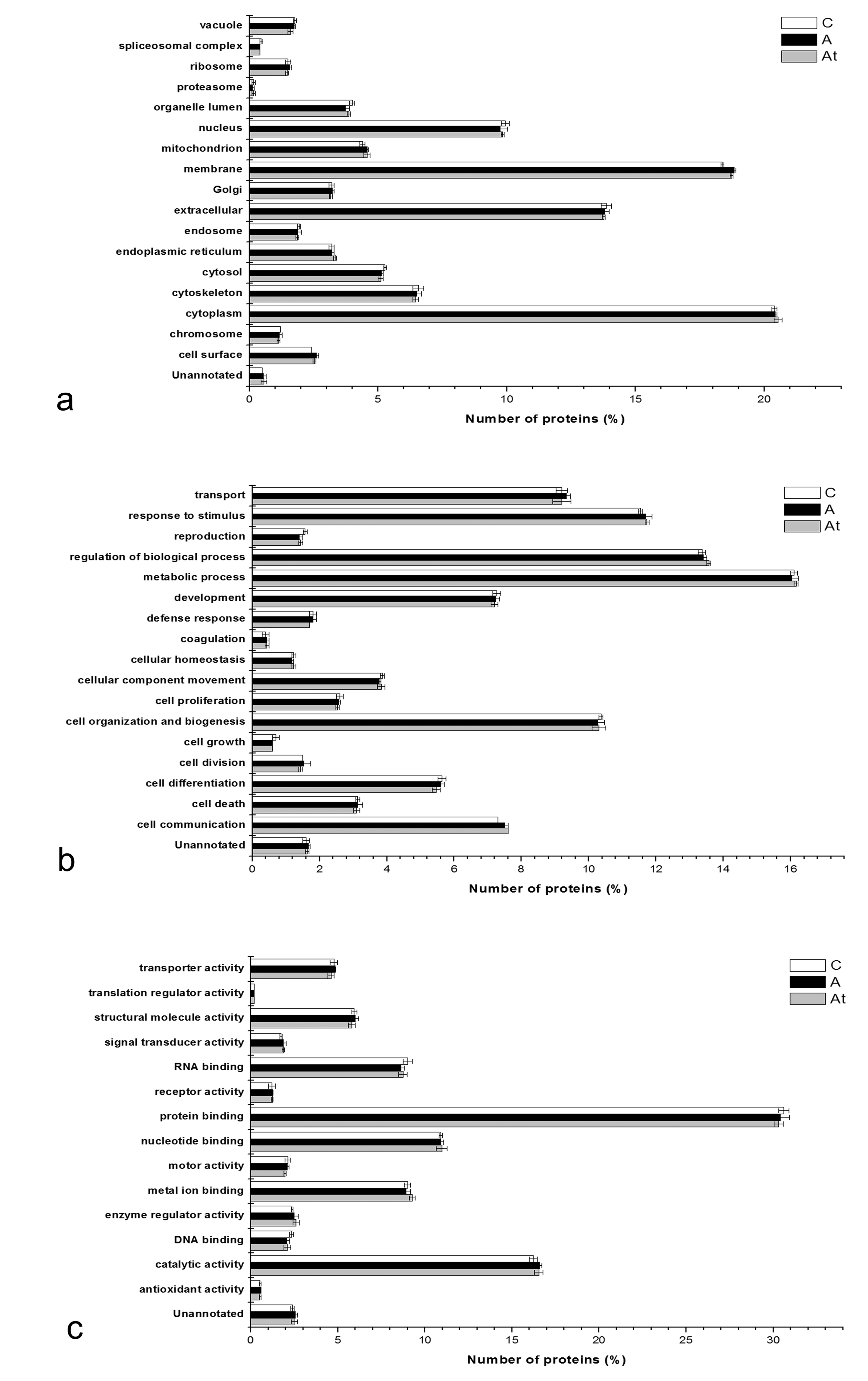


Additional file 4: Figure S2

Gene ontology details of detergent resistant membrane microdomains proteins. Data distribution based on: Cellular Component (a), Biological Process (b) and Molecular Function (c). Data expressed as mean values ± SD, following the analysis of the biological replicates
