# Supplementary material for: Alteration of actin dependent signaling pathways associated with membrane microdomains in hyperlipidemia
Source: Proteome Sci. 2015 Dec 1;13:30. doi: 10.1186/s12953-015-0087-0 (PMC4666118; doi:10.1186/s12953-015-0087-0)
Supplement: Additional file 6: Supplementary information. — (DOCX 10 kb) [file 12953_2015_87_MOESM6_ESM.docx]

Additional file 6: Supplementary information

Other proteins that are integrated into these three above mentioned signaling pathways that were identified by the present LC/MS-MS experiments, but were not found to be differentially expressed include: Alpha-actinin-4, Ras-related protein R-Ras and 2, Ras-related C3 botulinum toxin substrate 2, Cofilin-1, Monocyte differentiation antigen CD14, Dual specificity mitogen-activated protein kinase kinase 1, Cytoplasmic FMR1-interacting protein 1, Actin-related protein 2/3 complex subunit 1B and 3, Guanine nucleotide-binding protein G(I)/G(S)/G(O) subunit gamma-12, Myosin regulatory light chain 12B, Protein diaphanous homolog 1, Dedicator of cytokinesis protein, Integrin beta-3, Myosin light chain kinase 2, Laminin subunit alpha-4 and -5, Zyxin, Vitronectin, Collagen alpha-1 and 2 (including isoforms), Laminin subunit alpha-4, Myosin regulatory light polypeptide 9, Laminin subunit beta-2, Collagen alpha-2(I) chain, Alpha-parvin, Calpain-2 catalytic subunit, Ras-related protein Rap-1b.
